# Ghrelin and Glucagon-Like Peptide-1: A Gut-Brain Axis Battle for Food Reward

**DOI:** 10.3390/nu13030977

**Published:** 2021-03-17

**Authors:** Lea Decarie-Spain, Scott E. Kanoski

**Affiliations:** 1 Human & Evolutionary Biology Section, Department of Biological Sciences, University of Southern California, Los Angeles, CA 90089, USA; decaries@usc.edu; 2 Neuroscience Graduate Program, University of Southern California, Los Angeles, CA 90089, USA

**Keywords:** obesity, cue reactivity, GLP-1, meal anticipation, motivation, nutrient preference, flavor, aversion

## Abstract

Eating behaviors are influenced by the reinforcing properties of foods that can favor decisions driven by reward incentives over metabolic needs. These food reward-motivated behaviors are modulated by gut-derived peptides such as ghrelin and glucagon-like peptide-1 (GLP-1) that are well-established to promote or reduce energy intake, respectively. In this review we highlight the antagonizing actions of ghrelin and GLP-1 on various behavioral constructs related to food reward/reinforcement, including reactivity to food cues, conditioned meal anticipation, effort-based food-motivated behaviors, and flavor-nutrient preference and aversion learning. We integrate physiological and behavioral neuroscience studies conducted in both rodents and human to illustrate translational findings of interest for the treatment of obesity or metabolic impairments. Collectively, the literature discussed herein highlights a model where ghrelin and GLP-1 regulate food reward-motivated behaviors via both competing and independent neurobiological and behavioral mechanisms.

## 1. Introduction

The widespread availability of highly palatable and easily affordable food options in the modern era has substantially altered human eating behavior. The decision to eat, not to eat, and/or to select certain food items is largely regulated by hedonic and reward incentives rather than by energetic need. “Food reward” is a poorly defined, yet commonly used concept that generally refers to food-directed behaviors influenced by the potent reinforcing properties of certain food items, often rich in fat and sugar, that are highly palatable but also metabolically maladaptive. The hyper-reinforcing nature of such foods can contribute to the establishment and maintenance of food-motivated habits and lack of impulse control that can lead to metabolic dysfunction and/or obesity over the long term.

Food reward-motivated behaviors are potently influenced by gut-derived peptides that are released by cells of the gastrointestinal (GI) tract. In this review we focus on two such peptides, ghrelin and glucagon-like peptide-1 (GLP-1), that have opposing effects on energy consumption. Ghrelin is a stomach-derived hormone that increases appetite and food intake via its G protein–coupled receptor, type 1a growth hormone secretagogue receptor (GHSR1a) [1]. Circulating ghrelin levels are elevated during energy restriction and in anticipation of eating [2] and are decreased following a meal [3]. Glucagon-like peptide-1 (GLP-1) is a peptide secreted from L cells in the distal intestines and from neurons in the nucleus tractus solitarius (NTS) of the caudal brain stem. In addition to its well-known incretin effects, GLP-1 (and its FDA-approved analogs) potently reduces food intake via its G protein-coupled receptor, GLP-1 receptor (GLP-1R). Both ghrelin and GLP-1 affect food intake and metabolism via multiple biological pathways, including a paracrine vagus nerve pathway [4,5], blood-to-brain signaling, and in the case of GLP-1, projections from the GLP-1-producing preproglucagon neurons in the NTS throughout the neuraxis [6].

Several lines of emerging evidence highlight a role for both ghrelin and GLP-1 signaling in the mediation of food reward-motivated behaviors, and indeed, both systems are pharmaceutical targets for metabolic disorders. The present review addresses the competing influences of ghrelin and GLP-1 on various behavioral and neurocognitive appetitive domains, including reactivity to food cues, learned/conditioned meal anticipation, effort-based food motivated-behaviors, and flavor-nutrient preference and aversion learning. Preclinical rodent model studies reviewed herein emphasize sites of action in the brain mediating the influence of these two peptide systems on various food reward-associated behavioral domains. Studies investigating both endogenous and exogenous ghrelin and GLP-1 signaling in human participants are also reviewed, with an emphasis on the effects of bariatric surgery on these neurobiological processes.

## 2. Reactivity to Food Cues

Neural reactivity to cues associated with palatable food and the craving and behavioral responses that these cues elicit can be predictive of eating behaviors and propensity for weight gain [7]. Recent rodent model studies discussed herein reveal competing influences of ghrelin and GLP-1 on motivated responses to food reward-conditioned cues.

### 2.1. Rodent Studies

Results from rodent models reveal that ghrelin promotes food-directed behavior in response to a food-predictive cue. For example, oral gavage with the GHSR antagonist compound 26 in mice abolished food cue-potentiated eating [8]. Another study, however, reported that peripheral administration of a GHSR antagonist impaired the initiation of eating in a cue-potentiated eating procedure, yet had no effect on total sucrose consumed. The ventral subregion of the hippocampus (HPCv) may be a site of action mediating these effects, as rats trained to associate a conditioned stimulus (CS+) with palatable food availability consumed a larger number of meals immediately following CS+ presentation when they received ghrelin administration to the HPCv [9], an outcome that was not observed following presentation of stimuli not associated with food (CS−).

In addition to the HPCv, the VTA may be a brain region mediating ghrelin’s effects on food cue-stimulated eating. For example, in rats that had extinguished conditioned lever press responding for chocolate pellets, intra-VTA ghrelin administration facilitated the reinstatement of instrumental responding in response to a food reward-associated stimulus [10]. In contrast, however, another study reported that ghrelin infused directly into the VTA in rats did not influence lever pressing in response to food-associated cue presentation, although this same treatment increased appetitive lever pressing in a context with no stimuli presented [11]. More work is needed to further identify the specific brain regions, neural pathways, and behavioral parameters through which ghrelin potentiates eating and/or motivated responding in response to cues associated with palatable food.

Ghrelin may promote food cue-potentiated eating, in part, by acting as an indicator of fasting and/or food availability itself. For example, in the deprivation intensity discrimination task, where rats discriminate reinforcement outcomes based on food restriction-determined interoceptive state, either peripheral, intracerebroventricular (icv), or direct HPCv administration of ghrelin in food-sated rats mimics their conditioned appetitive behavior as if they had been food deprived for 24 h [12,13]. Ghrelin also alters baseline activity of dopamine neurons projecting from the VTA to the nucleus accumbens (ACB) via intraperitoneal (ip) injection in rats [14] or bath application on rat and mouse brain slices [15], an outcome similar to that occurring during fasting [16,17]. Alternatively, ghrelin signaling may also influence food cue reactivity by acting as a meal initiation signal. For example, neuronal firing patterns in the mediobasal hypothalamus of rats following the presentation of a cue predicting palatable food availability are replicated by an ip injection of ghrelin [18]. Finally, ghrelin may also affect cue-potentiated eating by enhancing the palatability of the reinforcer, an outcome consistent with findings that peripheral administration of a GHSR antagonist reduces sucrose palatability in mice as assessed via licking microstructure analyses [19].

While the effects of GLP-1 signaling on food cue reactivity in rodents have not been investigated as extensively compared to ghrelin, a recent study demonstrated that icv infusion of the GLP-1R agonist exendin-4 inhibits both phasic firing of ACB-projecting VTA dopamine neurons and food anticipatory approach behaviors in response to sucrose-predictive cues in rats [20]. In addition, infusion of exendin-4 into the paraventricular thalamus (PVT) in rats dampened the reinstatement of palatable food cue-induced lever pressing, as well as decreased the excitability of PVT neurons projecting to the ACB [21]. These findings suggest that GLP-1 reduces palatable food cue-induced food-seeking and eating by minimizing VTA and PVT input to the ACB. However, rodent model research on GLP-1 interactions with food cue-associated responses is thus far scarce, thereby highlighting a critical area for future preclinical investigation.

### 2.2. Human Studies

Functional brain imaging studies in healthy individuals provide additional evidence that ghrelin and GLP-1 have opposing effects on palatable food cue reactivity. For example, endogenous fasting levels of ghrelin predict activation of brain regions involved in reward processing in response to pictures of highly caloric and palatable food, including the caudate, amygdala, anterior cingulate, hippocampus, insula, and orbitofrontal cortex (OFC) [22,23]. In addition to these ghrelin-associated neural responses, plasma ghrelin concentrations can rise in response to viewing food pictures, suggesting a possible bidirectional relationship between ghrelin release and brain activity [24].

In the opposing direction, endogenous circulating concentrations of GLP-1 following consumption of a sugary beverage are negatively associated with dorsal striatum and OFC activation upon visual food cue presentation [25,26]. Further, in overweight participants following a weight loss program, postprandial GLP-1 levels along with dorsolateral prefrontal cortex (PFC) responsiveness to food cues were the best predictors of body mass index reduction [27]. Thus, interventions targeting food cue reactivity via GLP-1 signaling could be of interest for therapies targeting obesity.

In addition to the relationship between endogenous levels of ghrelin and food cue reactivity, administration of exogenous ghrelin modulates brain reactivity to food cues. For instance, subcutaneous (sc) injection of ghrelin in food-sated individuals increased OFC blood -oxygen-level dependent (BOLD) activity upon presentation of high energy food images, to an extent similar to the increased OFC BOLD response following an overnight fast [28]. In another group of healthy participants, intravenous (iv) ghrelin infusion potentiated activity in the ventromedial PFC to in response to abstract images (not food) paired to a food odor [29]. Interestingly, the same study revealed that functional connectivity between the hippocampus and the ventral striatum (containing the ACB) is increased by ghrelin and this is accompanied by improved performance in a reward prediction task. Administration of ghrelin (iv) also enhanced the BOLD responses in the hippocampus, VTA, insula, caudate, and amygdala in response to food cues, with responsiveness of the amygdala and OFC also being positively associated with self-reported hunger [30].

The actions of exogenous GLP-1 on brain reactivity to food cues have been largely investigated in obese and type 2 diabetic populations given the efficacy of long-acting GLP-1 analogs, such as liraglutide, in treating these metabolic conditions. For instance, obesity is characterized by greater insula responsiveness to food cues relative to normal weight individuals and this difference is abolished by acute treatment with the GLP-1R agonist exenatide [31]. Furthermore, the GLP-1-dependent suppression of insula reactivity to food cues predicted both a reduction in caloric intake [31] and lower scores for emotional eating [32]. In addition to the insula, obese individuals with type 2 diabetes show greater activation of the amygdala and OFC upon food cue presentation in a fasted state [32,33]. In a fed state, these effects are replicated by acute injection of the GLP-1R antagonist, exendin-9-39, especially in the type 2 diabetes group [33]. Chronic GLP-1 analog treatment also appears to alter food cue-induced brain activity responses, as 17 days of treatment with liraglutide in diabetic patients is sufficient to reduce insula and putamen responsiveness to highly desirable food cues, in conjunction with a decrease in hunger ratings in response to the food cues [34,35]. Interestingly, these changes occurred prior to any significant reduction in body weight, thus supporting a role for GLP-1 signaling in blunting reactivity to food cues independent of effects on body weight. However, in a subsequent cohort of diabetic patients treated for 5 weeks with liraglutide, activation of the OFC in response to food cues was increased, despite successful weight loss, improved metabolic profile, and reduced fat intake [36]. It remains to be determined whether this compensatory increase in OFC recruitment stems from negative energy balance, and/or whether this outcome impacts long-term weight maintenance.

Various bariatric surgery models are associated with acute improvements in metabolic function even prior to substantial weight loss (for review see [37]). Drastic change in circulating levels and amelioration of sensitivity to gut peptides such as ghrelin and GLP-1 likely contribute to the superiority of bariatric surgeries, such as Roux-en-Y gastric bypass (RYGB) and vertical sleeve gastrectomy (VSG), compared to dietary interventions alone in promoting sustainable weight loss [38,39]. Bariatric and dietary interventions may influence brain function differentially as bariatric surgery decreases resting-state functional connectivity between the precuneus and the insula, whereas dietary interventions increase it [40]. Thus, it may be that bariatric surgery promotes changes in neural activity that favor cognitive control over appetitive processes. For example, VSG surgery enhances resting-state functional connectivity between the hippocampus and the insula [41] and between the dorsolateral PFC and the anterior cingulate, with these outcomes accompanied by dampened cravings for high calorie foods [42] and reduced fasting levels of ghrelin [41,42]. These surgical induced changes in neural activity may influence food cue reactivity, as VSG surgery shifts attention from food to non-food cues, as well as reduces cravings and pleasantness ratings for food items [43]. Further, reductions in dorsolateral PFC BOLD response to food cues correlate with drops in fasting levels of ghrelin [44].

Similarly to the effects of GLP-1R agonism described above, RYGB surgery reduces insula and PFC BOLD signals in response to visual and auditory food cues in fasted obese women [45]. RYGB surgery also increases precuneus BOLD response to low fat/sugar food cues while decreasing response to high fat/sugar food cues [46]. Finally, weight loss and decreased appetite for sweet and fat foods after RYGB surgery coincides with reduced resting-state functional connectivity between the insula and the anterior cingulate as well as improved sensitivity of the default mode network to GLP-1R blockade [47].

### 2.3. Summary

Preclinical rodent model studies indicate that ghrelin signaling promotes conditioned appetitive behaviors and eating in response to food-associated cues, possibly by contributing to an interoceptive state analogous to fasting, by signaling meal anticipation, and/or by increasing the palatability of food. On the other hand, GLP-1 signaling may prevent cue-induced food-seeking by inhibiting neuronal input from the VTA and PVT to the ACB. These preclinical results are supported by functional neuroimaging analyses in humans revealing that circulating levels of ghrelin predict brain BOLD response to visual food cues while postprandial GLP-1 levels have opposite effects. Further, administration of exogenous ghrelin in food-sated individuals mimics the enhancement of brain BOLD response to food cues induced by fasting, whereas GLP-1R antagonism prevents the reduction of brain reactivity to food cues after consuming a meal. Short-term agonism of GLP-1R (via FDA-approved GLP-1 analog pharmacology) normalizes brain BOLD response to food cues in obese and diabetic individuals, although compensatory mechanisms may occur with longer duration of treatment. Similarly, bariatric surgery influences resting-state brain connectivity and dampens BOLD signal upon food cue presentation and these changes coincide with reduced fasting levels of ghrelin. Altogether, these results highlight the antagonizing actions of ghrelin and GLP-1 on brain reactivity to food cues and the potential therapeutic benefits of modulating these gut peptides.

## 3. Meal Anticipation

While gut peptide release is directly influenced by both long-term metabolic state and recent energy consumption, the learned anticipation of food availability is also sufficient to influence ghrelin and GLP-1 levels. For example, this is perhaps most dramatically illustrated by work from Woods and colleagues revealing that circulating levels of both GLP-1 and ghrelin are elevated prior to scheduled food access in meal entrained rodents [2,48]. Such endocrine responses may serve as a cephalic physiological primer for nutrient sensing and digestion. Conditioned meal-related factors can also influence postprandial endocrine levels, as meal anticipation potentiates postprandial suppression of circulating ghrelin in healthy men [49]. In this section we review rodent studies exploring the influences of ghrelin and GLP-1 signaling on food anticipatory activity and caloric intake in a scheduled feeding paradigm, as well as research in human participants measuring endocrine and neural outcomes in anticipation of eating.

### 3.1. Rodent Studies

Rodents on a restricted feeding schedule, with only a few hours of daily access to chow at a consistent time each day, rapidly develop a rise in locomotor activity in the few hours preceding food access, particularly activity directed towards the location of subsequent food access. This type of “food anticipatory activity” (FAA) is dampened in transgenic mice lacking either ghrelin or GHSR [15,50,51,52], thus supporting a role for ghrelin as a conditioned meal anticipation signal. Blunted FAA in GHSR knock-out (KO) mice is associated with decreased neural activation in the hypothalamus [52], VTA, and ACB shell [53]. Similar to cue-induced eating, the HPCv may be a critical site of action for ghrelin’s effects on meal anticipation as HPCv GHSR blockade reduced chow consumption in meal-entrained rats but had no effect on energy intake in non-entrained rats [54].

Ghrelin also appears to influence FAA based on scheduled access to palatable food in otherwise nonrestricted rats. For example, in rats with ad libitum access to chow, but predictable restricted access to high fat/sugar foods for a few hours daily, icv ghrelin increases FAA prior to the high fat/sugar meal and this effect is prevented by treatment with a GHSR antagonist [55]. The VTA may be a site mediating ghrelin effects on FAA for palatable food access, as intra-VTA ghrelin administration in rats entrained with access to a palatable high fat diet increases consumption in a GHSR dependent manner [56]. In contrast, another study reported decreased caloric intake of a high fat diet, but increased consumption of chow, following icv and VTA ghrelin infusion [57]. Although the contribution of ghrelin to FAA for standard chow is well established, additional work is required to further refine the role of ghrelin on FAA for palatable vs. bland/standard food access.

While the role of GLP-1 in food anticipatory behavior has not been as extensively investigated as ghrelin, GLP-1′s effects appear to oppose those of ghrelin. For example, infusion of the GLP-1R agonist exendin-4 into the ACB of rats with scheduled access to a high fat/sugar diet prevented the enhanced FAA and food consumption induced by mu opioid receptor agonism [58]. Paradoxically, however, both ghrelin and GLP-1 levels in circulation are increased prior to meal access in meal-entrained rats, as alluded to above in the work from Woods and colleagues. However, the temporal parameters are quite different. GLP-1 levels peak approximately 1 h before meal presentation and return to baseline prior to food presentation. Levels of ghrelin, on the other hand, peak approximately 30 min prior to food access yet remain substantially higher than baseline at the time of meal presentation [2]. GLP-1R antagonism prior to a conditioned spike in GLP-1 release reduced food intake at the subsequent food access period whereas iv infusion of a selective GLP-1R antagonist after the endogenous rise, but before food presentation, had the opposite effect [48]. While collectively these findings suggest that ghrelin and GLP-1 signaling have opposing effects on FAA and conditioned eating, more preclinical rodent work is needed to understand the functional role of premeal GLP-1 release, as well as the larger role of GLP-1 in meal anticipatory behavior.

### 3.2. Human Studies

Circulating levels of gut peptides in human participants fluctuate in accordance with the timing as well as the composition of an anticipated meal. For example, plasma ghrelin levels peak immediately prior to eating in individuals on a fixed intermeal interval schedule, suggesting that ghrelin primes meal initiation [59]. Interestingly, one’s individual habitual meal patterns appear to dictate the timing of a peak in fasting ghrelin prior to eating and this peak of ghrelin does not necessarily precede hunger [60]. These findings suggest ghrelin acts as a conditioned signal for meal anticipation rather than promoting hunger, per se. In addition to timing, preprandial concentrations of ghrelin can be influenced by the hedonic value of a meal. For instance, fasting levels of ghrelin are significantly higher in anticipation of consumption of chocolate versus an isocaloric non-palatable food item in obese male participants [61]. While less is understood about the fluctuation of endogenous GLP-1 level with regards to habitual eating patterns, there is evidence that exogenous GLP-1R agonist administration blunts FAA-associated neural responses. For example, iv infusion of the GLP-1R agonist exenatide dampens the heightened putamen, insula and amygdala BOLD responses in anticipation of chocolate milk delivery in diabetic individuals [62]. Thus, evidence to date is consistent with the notion that ghrelin and GLP-1 signaling have opposing effects on FAA and conditioned food consumption, and that these systems appear to interact with both the temporal and hedonic components of a meal.

### 3.3. Summary

Ghrelin signaling is strongly tied to FAA in rodent models of scheduled feeding, and these effects may involve signaling to the HPCv and VTA. GLP-1 appears to act via a more complex temporal relationship with conditioned feeding that warrants future studies, although there is evidence that FAA is blunted by ACB GLP-1R activation. In humans, the meal anticipatory rise in circulating ghrelin levels is adapted to both habitual meal patterns and the hedonic value of a meal, whereas GLP-1R agonism provides an avenue to normalize elevated brain reactivity in anticipation of palatable feeding.

## 4. Food Motivation

Willingness to work to obtain food is not surprisingly enhanced by a negative energy balance, but even under conditions energy repletion or sufficiency, food-motivated behaviors are influenced by gut peptides such as ghrelin and GLP-1. In rodents, motivation to seek out and/or work for food can be assessed in various conditioning tasks. For example, expression of a conditioned place preference (CPP) for a context previously paired with a food reward is indicative of food motivation in rodents, and when CPP compartment preference tests occur without food reinforcement, this procedure can assess food seeking in the absence of consumption. An alternative test is an appetitive operant conditioning task where the animal must press on a lever (or nose poke or other operant response) in order to obtain a food pellet, where the overall number of instrumental responses and the breakpoint (maximal number of presses the animal is willing to perform in order to obtain a reward in a progressive ratio schedule of reinforcement where cost of reward increases across the session) are measures of food motivation/willingness to work for food. Operant responding for food rewards can also be applied to human studies using handgrips or computer mouse clicks, for example.

### 4.1. Rodent Studies

Peripheral ghrelin administration appears to promote palatable food seeking behavior in rodents. For example, sc injection of ghrelin enhances CPP expression for a high fat diet in mice, whereas CPP expression is absent in GHSR KO mice or wildtype mice receiving acute oral gavage of the GHSR antagonist Compound 26 [63]. Similarly, blocking GHSR signaling with the GHSR antagonist, JMV2959, abolishes the expression of a CPP for chocolate in rats [64]. These effects are not mediated by the lateral parabrachial nucleus (lPBN) as infusion of ghrelin into this region has no impact on CPP for chocolate in rats [65]. Instead, ghrelin may interact with the hypothalamic neuropeptide orexin to drive CPP for palatable food given that CPP expression for a high fat diet is absent in orexin KO mice and in wildtype mice receiving ip injection of the orexin receptor antagonist SB-334867 [63].

In contrast, GLP-1R agonism blunts the expression of a CPP for palatable food in rodents. For example, administration of exendin-4 via ip injection in rats abolishes expression of a CPP for chocolate [66] and this suppression of CPP for palatable food is replicated with infusion of exendin-4 into the NTS [67,68] or the PVT [21], but not the HPCv [69]. This blunting of palatable food-motivated behavior may involve altered dopaminergic signaling as intra-NTS exendin-4 administration, in addition to blocking CPP expression, also increases gene expression levels for the catecholamine synthesis rate-limiting enzyme tyrosine hydroxylase (TH) and dopamine receptor type 2 in the VTA [68].

Ghrelin enhances willingness to work for palatable food in an appetitive operant conditioning task in mice following either sc [63,70] or ip [71] injections. On the other hand, GHSR antagonism via ip injection of JMV2959 reduces lever pressing for a 5% (weight/volume) sucrose solution in rats [72]. Central administration of ghrelin yields similar outcomes to peripheral injections, with an increase in operant responding for sucrose observed in rats following icv ghrelin injections [73,74]. Several pathways likely contribute to the central actions of ghrelin to promote instrumental responses for palatable food, with dopamine signaling from VTA to the ACB at the forefront. For example, VTA-ACB dopamine signaling is a critical for food-motivated behaviors [75], and early work from Abizaid and colleagues revealed that ghrelin binds to VTA neurons, where it increases dopamine turnover in the ACB in a GHSR-dependent manner [15]. Consistent with these results, at the behavioral level intra-VTA ghrelin administration increases lever pressing for sucrose [11,76] and flavored grain-based pellets [77,78]. Further, this influence of intra-VTA ghrelin requires dopamine signaling from the VTA to the ACB [76,78,79]. In addition, the increase in palatable food-motivated behaviors induced by ghrelin is prevented by administration of dopamine receptor type 1 and type 2 antagonists [76,78], thus further highlighting the critical role of downstream dopamine signaling. Ghrelin can also influence food motivation and ACB dopamine activity via sites of action other than the VTA. For example, infusion of ghrelin into the HPCv enhances operant responding for sucrose in rats, as well as increases phosphorylation of the dopamine synthesizing enzyme TH in the ACB [9]. The lateral hypothalamus (LHA) also partakes in the modulation of VTA to ACB dopamine signaling by ghrelin as intra-LHA ghrelin in rats increases lever pressing for sucrose [80] and potentiates dopamine release into the ACB upon pellet retrieval [81]. This could be mediated via interactions with hypothalamic orexin neurons as sc injection of ghrelin has no impact on operant responding for sucrose in orexin KO mice and wildtype animals treated with the orexin receptor antagonist SB-334867 [63]. In addition, infusion of ghrelin into the dorsal lateral septum (LS) increases lever pressing for sucrose in rats, although GHSR blockade into the dorsal LS does not influence operant responding [82]. Finally, the lPBN does not appear to participate in the motivational properties of ghrelin as direct infusion into this nucleus has no impact on lever pressing for sucrose [65].

As opposed to ghrelin, GLP-1 signaling reduces instrumental responding for high fat/sucrose food rewards. Administration of exendin-4 in rats, for example, whether via ip injection [66,83,84] or icv infusion [66,85], dampens motivated lever pressing for sucrose. As a primary CNS node in the gut-to-brain axis, it is not surprising that the NTS contributes to the central effects of GLP-1 signaling on food motivated behaviors. In fact, the inhibition of lever pressing induced by central administration of exendin-4 is replicated in rats by specific infusion into the NTS [67,68,86], suggesting that the NTS may be mediating the effects of peripheral GLP-1 analog administration on effort-based food-motivated behavior. Several other brain structures also partake in the central actions of GLP-1 signaling on food-motivated operant responding, either via direct projections from the NTS or potentially GLP-1 volume transmission signaling through the cerebrospinal fluid. For example, operant responding for palatable food in rats is reduced by infusion of exendin-4 or GLP-1 into either the VTA or ACB [66,83], PVT [21], HPCv [69], lPBN [87], dorsal LS [88] and supramammillary nucleus (SuM) or LHA [86]. Further, intra-PVT administration blunts the excitability PVT projecting towards the ACB [21], once again evidencing a pivotal role for the ACB and mesolimbic dopamine signaling in GLP-1′s role in food motivation. However, dopaminergic activity in other limbic structures can also interact with GLP-1 as, in addition to reducing lever pressing for sucrose, icv exendin-4 enhances dopamine turnover in the amygdala and these effects are partly inhibited by intra-amygdala infusion of a dopamine receptor type 2 antagonist [85].

While there is a consensus in the literature for the opposing effects of ghrelin and GLP-1 signaling on food-motivated behaviors, studies including both male and female rodents shed light on sex-specific mechanisms of action [89]. For example, LHA infusion of ghrelin increases lever pressing for sucrose in both male and female rats, but intra-LHA administration of the GHSR antagonist YIL781 only inhibits operant responding in females [80]. The same group observed that GLP-1R agonism with exendin-4 in the LHA decreases lever pressing for sucrose in both sexes, but GLP-1R blockade enhances operant responding only in male rats [90]. These results suggest that the endogenous relevance of LHA GHSR signaling to palatable food-motivated responding is more prominent in females, whereas the opposite is true for LHA GLP-1 signaling. Additional work highlighted the SuM as a site of action for exendin-4 to diminish lever pressing for sucrose in males only, whereas infusion into the VTA reduced food-directed lever pressing in both sexes, but more potently in females [91]. In addition, the inhibitory effect of intragastric nutrient infusion on food-motivated behaviors may operate via sex-specific pathways as ip injection of the GLP-1R antagonist exendin-9 prevents the reduction in operant responding following nutrient infusion in male but not female rats [92]. Although the general opposing behavioral outcomes related to food-motivated behavior for the GLP-1 and ghrelin systems are similar across sexes, the specific underlying mechanisms mediating neural sites of action can diverge.

Impulsivity, or responding without apparent forethought for the consequences of one’s actions, and inhibitory control, or the ability to exert “top-down” control over motivated responses, are key psychological constructs related to food-motivated responding. Ghrelin and GLP-1 have each been associated with impulsivity and inhibitory control. For example, ghrelin can promote food motivated behaviors by driving impulsivity as both icv and VTA infusion of ghrelin increases the rates of impulsive responding in rats assessed in tasks of differential reinforcement of low rates of responding (DRL), go/no-go, and delay discounting [93]. On the other hand, infusion of exendin-4 into the HPCv of rats inhibits impulsive responding for high fat/sucrose pellets in the DRL task [94]. In addition, GLP-1 signaling may limit impulsivity by promoting inhibitory control. For example, daily ip injections with the GLP-1 analog, liraglutide, over 12 days potentiates inhibition of conditioned appetitive responses as rats limit food-seeking behavior in response to a negative feature stimulus that signals when a target stimulus will not be followed by food reinforcement [95]. Thus, food-motivated behavioral responses can be modulated via ghrelin’s and GLP-1′s influences on impulsivity and inhibitory control. In some cases, impulsive behavior can be observed following treatments that do not also affect food-motivated operant responding [96], and thus it is critical for future work to determine whether the effects of ghrelin and GLP-1 signaling on impulsivity and/or inhibitory control are linked with vs. distinct from the general effects on food-motivated operant responding.

### 4.2. Human Studies

GLP-1′s influence on food-motivated behaviors in human participants can be impaired by metabolic conditions. For example, in an incentive motivation task where participants exert physical strength on a handgrip to obtain food or monetary rewards, sc injection of the GLP-1 analog liraglutide prevents the increase in incentive motivation induced by hunger in participants with high, but not low, insulin sensitivity [97]. Interestingly, RYGB surgery reduces computer mouse clicks for a sweet and fatty candy [98] and this may be related to improvements in GLP-1 signaling. In fact, indirect inhibition of GLP-1 secretion via sc injection of octreotide, a somatostatin analog, enhances computer mouse clicks for chocolate rewards in individuals who underwent RYGB surgery but not in obese non-operated controls, suggesting that RYGB surgery influences the effects of GLP-1 signaling on food-motivated responses [99]. Similarly, patients who underwent esophagectomy for cancer present elevated postprandial levels of GLP-1 relative to healthy control subjects and also present an increase in computer mouse clicks for chocolate following sc injection of octreotide, whereas healthy controls do not [100]. Thus, the extent to which GLP-1 signaling dampens food motivation is influenced by chronic metabolic status. To our knowledge the effects of ghrelin signaling on food-motivated responding has thus far received limited attention, however, based on the wealth of preclinical data discussed above it is highly probable that ghrelin’s effects oppose those of GLP-1 in humans as well.

### 4.3. Summary

Ghrelin and GLP-1 compete to enhance and inhibit the expression of effort-based food-motivated behaviors, respectively. Rodent model studies identify several brain regions through which ghrelin and GLP-1 act to influence CPP expression and operant responding for food, with various neuronal outputs that likely converge onto the ACB. Despite similar behavioral outcomes across sexes, the specific mechanisms and sites of action underlining ghrelin and GLP-1′s actions on food motivation can be sex-specific. In addition, food-motivated responding can be modulated by ghrelin and GLP-1, at least in part, via their opposing impacts on impulsivity and inhibitory control. Finally, the contribution of GLP-1 signaling to regulate food motivated behaviors in humans is influenced by metabolic parameters such as insulin resistance and bariatric surgery.

## 5. Nutrient Preference

Palatable foods are typically rich in fat and/or sugar and tend to be preferred over low fat/sugar items. The magnitude of this preference, however, can be influenced by gut peptides such as ghrelin and GLP-1. In this section, we review studies measuring free intake of palatable foods in rodents and human participants that vary with regards to macronutrient content, palatability, and other factors.

### 5.1. Rodent Studies

Ghrelin signaling enhances preference for palatable foods, especially sweetened solutions, even if nonnutritive. For example, ip injection of ghrelin enhances consumption of a 0.1% (*w*/*v*) saccharin solution in mice [101] and of a 5% (*w*/*v*) solution in rats [72], while GHSR antagonism with ip injection of JMV2959 reduces intake of a 2% (*w*/*v*) sucrose solution in prairies voles [102] and of a 0.1% (*w*/*v*) saccharin beverage in mice [72]. In addition, relative to wildtype controls, preference for a sweetened solution is dampened in transgenic mice lacking ghrelin [103] or GHSR [104]. One study, however, observed that icv infusion of ghrelin does not influence intake of a 5mM saccharin solution in rats [105]. Additional work on the central actions of ghrelin to enhance intake of sugar and other sweet tastants is required in order to elucidate if this finding is related to the route of administration and/or other variables. In contrast, the effects of ghrelin on preference for high fat foods are less consistent across studies. For example, when rats are offered unlimited access to diets enriched in fat, carbohydrate, or protein, intra-VTA infusion of the GHSR antagonist GHRP over 14 days specifically lowers fat intake [77]. Similarly, mice lacking GHSR and rats injected (ip) with JMV2959 show reduced preference for palatable foods and intra-VTA ghrelin increases intake of peanut butter, but not standard chow [64]. However, rats offered ad libitum access to lard and standard chow increase their intake of both foods following icv infusion of ghrelin, but in contrast to the previous study, only increase their chow intake when ghrelin is infused into the VTA [106]. This specific increase in chow, but not lard intake is also reported in rats receiving ghrelin infusion into the lPBN [65].

In opposite to ghrelin, GLP-1 agonism appears to reduce caloric intake by preferentially targeting consumption of both fat and sugar-rich palatable foods. For example, ip injection of GLP-1 reduces intake of sucrose [107] but not standard chow in mice, at least under the conditions tested in this study [105]. In addition, infusion of exendin-4 into either the VTA or the core subregion of the ACB decreases sucrose intake in rats while consumption of standard chow is unaffected [108]. Similarly, exendin-4 injections into the NTS of rats decreases intake of peanut butter but not standard chow [68] and acute infusion into the 4^th^ ventricle of an inhibitor of the enzyme degrading GLP-1 reduces intake of a high-fat diet and not chow [109]. Interestingly, this preferential reduction of palatable food intake is observed in the few hours following exendin-4 infusion and is followed by a generalized reduction in caloric intake. For example, rats administered exendin-4 into the HPCv only decrease their consumption of a high fat/sugar diet 6h after the infusion, but at the 24h timepoint chow intake is also reduced [69]. This is similar in rats receiving exendin-4 into the SuM, where only sucrose intake is inhibited 1h after treatment, whereas sucrose, fat and standard chow consumption are all lower after 24h [91]. These findings suggest that GLP-1R agonism could acutely preferentially reduce intake of palatable food prior to exerting a generalized decrease in food intake. Consistent with this framework, daily sc injections of liraglutide over 25 days reduce overall caloric intake similarly across macronutrients in rats offered a cafeteria diet [110].

### 5.2. Human Studies

Ghrelin and GLP-1 are differentially influenced by the consumption of palatable food. For example, postprandial levels of ghrelin are significantly higher after consuming a pleasurable food item, such as a pastry, compared to after eating an isocaloric portion of bread and butter [111,112]. Ghrelin may even contribute to genetic predispositions for sweet taste preference as in a Swedish population cohort, specific haplotypes for the pro-ghrelin gene are associated with an elevated consumption of sucrose, especially in men [72]. In contrast, fasting GLP-1 concentrations negatively predict intake of food rich in simple sugars in a vending machine paradigm [113], which the authors interpreted as evidence that GLP-1 plays a role in reward pathways regulating simple sugar intake. A number of studies also report altered food preferences following gastric bypass surgery, with a shift away from high sugar/fat preference [114,115,116,117]. However, the extent to which these surgical-induced changes in food preference are either directly or indirectly influenced by altered post-surgery ghrelin and/or GLP-1 levels requires future investigation.

### 5.3. Summary

Palatable food intake, especially the consumption sweetened solutions, is enhanced by ghrelin, whereas GLP-1 preferentially reduces intake of high fat and high sugar foods, at least following acute administration. In addition, work in both rodent and humans reveals that preference for fat and sugar can be altered by bariatric surgery and contribute to weight loss, yet linking these effects to altered ghrelin or GLP-1 signaling remain to be established. Finally, circulating levels of ghrelin and GLP-1 can be indicative of palatable food consumption in humans.

## 6. Flavor

Flavor perception contributes substantially to the reinforcing properties of palatable food and learned associations between flavor and postingestive outcomes can influence future eating behaviors. In this section, we review ghrelin- and GLP-1-relevant rodent model studies assessing licking patterns in response to escalating doses of sucrose solutions or lipid emulsion, as well as orofacial reactions to oral infusions of rewarding or aversive solutions. In addition, we discuss changes in flavor sensitivity and endocrine response to orosensory stimulation in humans. 

### 6.1. Rodent Studies

Work in transgenic mice lacking essential component for ghrelin signaling identify ghrelin’s contribution to flavor perception. For example, the distribution of licks in response to increasing concentrations of sodium chloride or citric acid reveals reduced sensitivity to the aversive properties of highly concentrated salty and sour solutions in GHSR KO mice [118]. Similarly, mice lacking ghrelin O-acyltransferase (GOAT), the enzyme generating the active form of ghrelin, display dampened sensitivity to escalating concentrations of sodium chloride, in addition to lower licking responses for high doses of intralipid [119]. These differences in licking responses are associated to altered protein expression for a salt-sensitive subunit in GHSR KO mice [118] and fatty acid receptors in GOAT null mice [119], thus further highlighting the contribution of ghrelin signaling to salty and fatty taste perception.

While genetic ablation of ghrelin signaling appears to make salty tastants less aversive, GLP-1 receptor KO mice on the other hand demonstrate blunted increases in licking for escalating doses of palatable sweet solutions (both sucrose and sucralose solutions) relative to wildtype animals [120,121,122]. Reduced responsivity to high doses of sucrose is also reported from gustatory nerve recordings in GLP-1R KO mice [122]. In addition to sweet taste, GLP-1 receptor KO mice display greater increases in licking for high doses of umami tastants [121] and a stronger decreased oral response to citric acid [120]. The implication of GLP-1 signaling in the orosensory component of a meal is reinforced by findings that icv infusion of GLP-1 reduces sucrose intake even in rats with gastric cannulas, preventing intestinal nutrient sensing [123]. Interestingly, mice infused with exendin-4 into the dorsal LS present a decrease in licking rate for a 0.25 M sucrose solution whereas licking microstructure is unchanged [88]. In addition, rats receiving ip injection of exendin-4 demonstrate unaltered orofacial reactivity to sucrose, indicating no changes in the hedonic response, yet they also show reduced aversive orofacial responses to a quinine solution [124]. Thus, GLP-1 signaling appears to dampen licking responses to high concentrations of sweet tastant, but not by altering the hedonic properties of sweet taste perception.

### 6.2. Human Studies

Ghrelin is modulated by orosensory stimulation even in the absence of nutrient absorption. For example, sham feeding studies, where food is smelled, chewed and tasted, but not swallowed, reveal reductions in circulating ghrelin to the same extent as induced by food consumption [125]. Further, despite lack of nutrient sensing in the GI tract, circulating levels of ghrelin are higher after a sham feeding session with a meal enriched in protein rather than carbohydrates or fats [126], solidifying the notion that ghrelin is tied not only to nutrient sensing but also orosensory flavor processing.

Interventions targeting obesity have the potential to alter taste perception, potentially via GLP-1 signaling. For instance, obese women present dampened sweet taste sensitivity when compared to lean individuals [127]. However, taste perception of low concentrations of sucrose is improved following RYGB surgery [128], although these findings are yet to be directly linked to post-surgical changes in circulating GLP-1. In addition, obese subjects chronically treated with liraglutide show a reduction in their preferred concentration of sweet, salty, savory and fatty-flavored solution after 16 weeks, while these preferences remain unchanged in the placebo group [129]. Similarly, GLP-1R agonism over 3 months for the treatment of type 2 diabetes improves sweet taste sensitivity and decreases preferred concentrations for a lipid emulsion [130]. The amplitude of such effects of GLP-1 signaling may be sex-dependent as, in a food choice task based on flavor, women were more likely than men to change their flavor preferences after a GLP-1 iv infusion [131].

### 6.3. Summary

Ghrelin and GLP-1 signaling contribute to the perception of different flavors, even in the absence of GI nutrient sensing. Enhancement of GLP-1 signaling has the potential to influence food preferences and improve taste sensitivity, especially the detection of low concentrations of sucrose. Consistent with our larger framework that ghrelin and GLP-1 have opposing effects on processes related to food reward, both ghrelin loss of function (transgenic KO models) and GLP-1 gain of function (GLP-1 analog treatment) appear to reduce taste sensitivity to chloride-based solutions, although more research is needed to directly compare the two systems on various domains of flavor processing.

## 7. Aversion

Eating patterns can be potently shaped by aversive experiences such as malaise, nausea, or emesis that function to discourage future consumption in a flavor-specific manner. Not surprisingly based on their competing influences on gastric motility, ghrelin and GLP-1 appear to distinctively influence GI-associated malaise. Pharmaceutical agents that decrease food intake, including FDA-approved GLP-1 analogs, often concomitantly promote GI discomfort, thus limiting their clinical application by developed tolerance to these aversive effects. In this section, we review rodent model studies examining the impacts of ghrelin and GLP-1 on conditioned flavor avoidance (CFA) where a neutral flavor is paired to an ip injection of some compound that may produce nausea (e.g., lithium chloride), as well as on pica, the consumption of non-nutritive items such as kaolin as a means to relieve nausea. Specifically, we explore the contribution of aversion and nausea to the dampening actions of GLP-1 signaling on food motivation and we discuss the clinical potential for ghrelin to alleviate nausea. 

### 7.1. Rodent Studies

Ghrelin administration attenuates CFA induced by lithium chloride, at least in part, by acting specifically in the lateral amygdala in rats. For example, while ip injection of the GHSR antagonist JMV2959 alone has no impact on CFA [132], infusion of ghrelin directly in the lateral amygdala prevents the acquisition of a lithium chloride-induced CFA in a GHSR-dependent manner [133,134] but also its extinction [135]. In addition, GHSR signaling may provide an avenue to reduce food intake without promoting CFA as treatment with novel GHSR inverse agonists inhibits standard chow intake without promoting CFA in mice [136]. Further, exogenous ghrelin could be beneficial for the relief of nausea associated to medical treatment. Rats receiving the chemotherapy agent cisplatin present dampened ghrelin presence in the hypothalamus [137] and increased gene expression for GHSR in this same region [138], suggesting that these effects could be attenuated by exogenous ghrelin. In fact, ip injection or NTS infusion of ghrelin reduces nausea-related pica by improving gastric motility in rats treated with cisplatin [139,140]. In addition, the GHSR agonist HM01 reduces emesis induced by motion or cisplatin in the shrew [141].

In contrast, GLP-1 signaling delays gastric emptying and the resulting GI discomfort may contribute to GLP-1s capacity to stimulate nausea and conditioned aversion. Pairing an icv infusion of GLP-1 to a neutral flavor is sufficient to induce CFA [142,143] and ip injection of either exendin-4 or liraglutide promotes CFA in a dose-dependent manner in rats [144,145]. The aversive properties of GLP-1 signaling may be dependent on the route of administration, as infusion of GLP-1 into the hepatic portal vein [146], PVN [147], ACB [148], lateral dorsal tegmental nucleus [149] or even the lateral ventricle [150] inhibits food intake without inducing CFA or pica in rats. It has been suggested that the vagus nerve mediates the aversive effects of GLP-1, as ip injection of exendin-4 induces CFA in vagotomised but not sham-operated rats [151]. However, additional work reveals that systemic administration of extendin-4 at various doses induces both CFA and kaolin consumption, and in both vagotomised and sham-operated rats [152]. Further, the authors identify the NTS as the most likely candidate for the malaise induced by GLP-1 signaling [145]. Others, however, have found reductions in CPP expression and lever pressing for palatable food following intra-NTS exendin-4 at doses that do not also produce pica [67,68]. In addition to not assessing CFA, these studies differ from the previous findings by usage of a shorter habituation period to the kaolin pellets [68] or lower dose of intra-NTS exendin-4 [67]. Consistent with a role for NTS GLP-1R signaling in mediating the nausea outcomes induced by this system, GLP-1R blockade attenuates anorexia and weight loss when infused into the NTS of tumor-bearing rats [153], and reduces pica induced by icv infusion in cisplatin-treated rats treated [154].

Although the literature point towards aversive responses to GLP-1, some studies have still found reductions in food reward-motivated behavior by brain site-specific GLP-1R agonists using doses that were not associated with CFA and/or pica. For example, exendin-4 or GLP-1 has been shown to reduced effort-based food-motivated behaviors in rats without influencing CFA or pica when administered into the VTA [66,108], PVT [21], HPCv [69], lPBN [87] and SuM [86]. Infusion of GLP-1R agonists in these brain nuclei possibly targets the neural mechanisms underlining food motivation and bypasses the hindbrain GLP-1 nausea circuitry. These findings overall suggest that GLP-1 signaling can inhibit the palatable food-motivated behaviors by mechanisms other than GI discomfort based on analyses from site-specific brain region application.

### 7.2. Human Studies

Changes in ghrelin signaling are tied to GI discomfort across various health conditions in humans. In healthy participants, for example, susceptibility to motion sickness is associated to a sudden drop in circulating levels of ghrelin [155]. Patients treated with cisplatin also present lower concentrations of circulating ghrelin [156] and GHSR agonism alleviates nausea and vomiting induced by chemotherapy [157,158,159,160]. In addition, GHSR agonists can provide relief to diabetic individuals experiencing nausea and vomiting as a result of gastroparesis [161,162,163,164]. Thus, ghrelin signaling may promote food reward-motivated behaviors in humans, in part, by minimizing GI discomfort.

Conversely, the most common side effects associated with treatment for obesity and type 2 diabetes with GLP-1 analogs are nausea and vomiting. These symptoms may be more frequent in women and those also treated with metformin, proton pump inhibitors, and/or anti-histamines [165,166]. While nausea and vomiting associated with weight loss from liraglutide treatment is often transient [167], occurrence of these side effects still limits the therapeutic potential of GLP-1 analogs. A recent study demonstrates that combination of a lower dose of GLP-1 analog with insulin therapy improves glucose homeostasis and reduces the prevalence of GI symptoms, which thus could strengthen treatment adherence [168]. Another therapeutic option is the development of site-specific GLP-1 analogs such as exendin-4 conjugated to vitamin B12 resulting in limited brain penetrance to improve glucose homeostasis without the effects on appetite [169,170]. Moreover, pharmacological targeting of the neural pathways underlining the actions of GLP-1 signaling on food reward without concomitant nausea could promote weight loss and bypass aversive side effects.

### 7.3. Summary

Ghrelin alleviates GI discomfort while the malaise induced by GLP-1 can drive CFA and pica in rodents. However, rodent model studies reveal that reductions in food reward can be obtained with exendin-4 in the absence of aversive GI effects based on site-specific brain application. The development of improved GLP-1 analogs could ultimately promote weight loss without inducing nausea and vomiting in obese individuals, although clinical use of effective GLP-1 analogs absent of nausea side effects is thus far absent. Overall, the evidence suggests that the opposing effects of ghrelin and GLP-1 on mediating GI-associated malaise may be a driving component of their opposing effects on food reward-motivated behaviors.

## 8. Future Directions

This review summarizes the antagonizing actions of ghrelin and GLP-1 on distinct food reward-associated behavioral constructs. While basic science knowledge of each of these systems has increased dramatically in the past decade, there are gaps in the literature that, if addressed, could provide a more complete neurobiological and behavioral framework that translates to the clinical setting. For example, additional preclinical work on the role of GLP-1 in reactivity to food cues and meal anticipation would be beneficial given that the majority of rodent model studies focusing on these behavioral domains assessed the contributions of ghrelin and other orexigenic signals (e.g., orexin/hypocretin). In addition, recent advances in real-time in vivo calcium-based neuronal recording in rodents offer an opportunity to bridge findings from human brain imaging studies towards a deeper understanding at the levels of cell type specificity and systems neuroscience. Finally, although the alterations in ghrelin and GLP-1 signaling following bariatric interventions have been widely investigated, the direct causal contributions of these physiological changes to alterations in food reward-associated behaviors remain to be determined.

## 9. Conclusions

**Figure 1:**
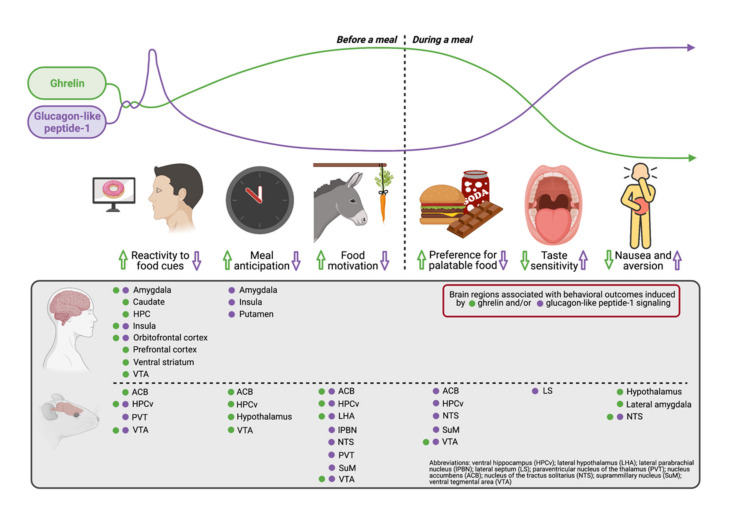
Overview of ghrelin (green) and glucagon-like peptide-1 (purple) circulating levels in relation to a meal (**top row**) and the competing actions (↑increase or ↓decrease) of these systems on distinct behavioral domains related to food reward (**middle row**) and associated sites of actions based on human and rodent model results (**bottom row**).

Ghrelin and GLP-1 act as opposing forces on multiple behavioral, physiological, and neural fronts related to obtaining and consuming highly palatable and “rewarding” foods (summarized in Figure 1). For example, prior to feeding, ghrelin promotes whereas GLP-1 dampens brain activity in response to food cues. In addition, meal anticipation is largely driven by endogenous ghrelin, particularly in anticipation of palatable food access. Similar to fasting, ghrelin enhances various palatable food-motivated effort-based responses, whereas GLP-1 has opposing effects via action in multiple sites across the neuraxis. The preference for high fat/sugar foods is also heightened by ghrelin and reduced by GLP-1 signaling. These changes may be attributed, in part, to alterations in flavor perception, and/or modulation of GI-associated nausea and malaise.

We conclude by noting that the opposing influences of ghrelin and GLP-1 signaling on these various food reward-associated behavioral constructs (food cue reactivity, meal anticipatory activity, palatable food-motivated responding, altered food preferences, flavor, and/or GI-associated malaise) likely occur with varying degrees of mutual exclusion. In other words, it is likely that modulation of one food reward-associated construct by ghrelin or GLP-1 may be secondary to effects on a separate construct. For example, altered orosensory and retronasal flavor processing, and/or altered GI-malaise processing, is likely to subsequently influence food-motivated responses, reactivity to food cues, nutrient preference, etc. Generally, however, the literature suggests that ghrelin and GLP-1 have opposing effects on these food reward-associated behaviors via both distinct and overlapping neurobiological and behavioral mechanisms. Understanding the extent that these (and other) gut peptide systems concurrently but also independently affect these food reward-associated processes will require sophisticated behavioral analyses to carefully dissect these distinct constructs.
